# Natural Products for Interventions for Chronic Diseases: From Source to Therapy

**DOI:** 10.3390/biomedicines14071507

**Published:** 2026-07-03

**Authors:** Qilei Chen

**Affiliations:** 1 Teaching and Research Division, School of Chinese Medicine, Hong Kong Baptist University, Hong Kong 999077, China; qlchen@life.hkbu.edu.hk; 2 Institute for Research and Continuing Education, Hong Kong Baptist University, Shenzhen 518000, China

## 1. Introduction

Chronic diseases are the foremost global health challenge of our era, with an immense burden driven by cardiovascular disorders [1], cancer [2], and a spectrum of metabolic and inflammatory conditions [3]. While conventional pharmacotherapy has made significant strides, its effectiveness is often hampered by adverse side effects, the emergence of acquired resistance (particularly in oncology) [4], and the complex, multifactorial nature of these ailments [5]. This clinical reality necessitates a continuous search for novel therapeutic strategies [6]. In this context, natural products have re-emerged at the forefront of scientific investigation [7]. Long serving as a rich reservoir for drug discovery, these compounds are now being explored with unprecedented precision, moving beyond their empirical use and becoming cornerstones of evidence-based, mechanism-driven medicine [8].

This Special Issue, “Natural Products for Interventions for Chronic Diseases: From Source to Therapy”, was conceived to capture the dynamism of this field. We are pleased to present a collection of 11 outstanding articles—comprising original research and comprehensive reviews—that collectively map the scientific journey of natural products. The contributions herein span the full spectrum of drug discovery: from identifying the natural source and elucidating the molecular mechanism to exploring preclinical therapy. Together, they paint a compelling picture of a field that is increasingly precise, targeted, and clinically relevant.

## 2. A Spotlight on Bioactive Compounds: From Broad Potential to Specific Applications

The journey often begins with a comprehensive understanding of a single plant or compound, as perfectly exemplified by two broad-ranging reviews in this Special Issue. One review, led by Villegas-Vazquez, unveils the multifaceted role of *Moringa oleifera*, the “miracle tree” native to India, in managing a wide array of chronic diseases, showcasing its rich phytochemical profile and diverse pharmacological actions [9]. Complementing this holistic perspective, another review by Leong charts the therapeutic potential of a specific stilbenoid, pterostilbene, in treating cardiovascular diseases, detailing its journey from molecular mechanisms to its effects on diverse preclinical models [10].

Building on this broad perspective, several articles in this issue drill down into the specific applications of individual compounds. An outstanding example is the work by Zhang et al., who demonstrate that the flavonoid kaempferol can alleviate glucocorticoid-induced osteonecrosis. They meticulously show how it modulates macrophage M1/M2 polarization through the targeted activation of RhoA/ROCK-mediated mitophagy [11]. In another highly specific study, Li et al. show that ferulic acid can mitigate chemotherapy-induced premature ovarian insufficiency by targeting the Grp78 and Perk-eIF2α-ATF4-CHOP pathway to attenuate endoplasmic reticulum stress [12]. Further expanding the therapeutic scope, Stevens Barron et al. explore the synergistic and antagonistic effects of vitamin E isoforms in cancer cells, offering key insights for combination therapies [13].

## 3. Mechanism-Driven Discovery: Targeting Key Pathways and Novel Players

A defining feature of modern natural product research, which is strongly represented in this issue, is the shift from phenomenological observation to a mechanism-driven discovery paradigm. This is powerfully illustrated by a systematic review by Huang and Wan, which synthesizes the preclinical evidence for targeting the AMPK pathway in heart failure [14]. Their work showcases how a central metabolic regulator can serve as a convergent node for diverse therapeutic interventions using natural compounds.

This collection also advances the scientific frontier by exploring emerging therapeutic targets and novel intervention strategies. One forward-looking review moves “Beyond PAD Inhibition” to provide a strategic roadmap for targeting citrullination, a post-translational modification increasingly implicated in autoimmune diseases [15]. Instead of focusing solely on enzyme inhibition, this work conceptualizes a multi-layered approach. It outlines how natural products can act not only as direct PAD inhibitors but also as indirect modulators of upstream signaling or, most innovatively, as substrate-centric protectors that shield key arginine residues from pathogenic modification. These contributions underscore a critical evolution in the field: the focus is no longer just on what a natural product does but on how it achieves its effects with molecular precision.

## 4. Expanding the Therapeutic Horizon: From Traditional Sources to Innovative Interventions

The diversity of this Special Issue is reflected in the breadth of sources and applications it covers. A review by Dinić et al. provides a panoramic survey of the plant family Gentianaceae, summarizing its therapeutic potential in the treatment of diabetes and its complications [16]. This work bridges traditional ethnopharmacological knowledge with modern scientific validation. At the same time, Lee et al. offer a focused investigation into an aqueous extract of *Piper sarmentosum*, demonstrating its ability to mitigate osteoarthritic pathology in human chondrocytes by enhancing anabolic activity and attenuating NO-driven catabolism [17].

This issue also embraces novel and thought-provoking concepts of “intervention”. The study by Kumar et al. on short-term post-traumatic high-sucrose intake offers a nuanced look at how a dietary component can attenuate acute fear responses in mice, expanding our understanding of how “natural” interventions can modulate neuropsychiatric phenomena [18]. Finally, connecting the dots on the path to therapy, a systematic review by Galant et al. addresses the critical challenge of drug administration [19]. By analyzing randomized clinical trials on percutaneous drug delivery to the masticatory system, this work highlights a crucial, and often overlooked, aspect of translating a bioactive compound into an effective therapy: ensuring it reaches its target tissue.

## 5. Conclusions and Future Perspectives

Collectively, the 11 articles in this Special Issue articulate a clear and powerful message: natural product research has evolved from a field of serendipitous discovery into a paradigm of rational, mechanism-driven science. The journey from a natural source to a therapeutic is now illuminated by molecular precision, as demonstrated by the diverse studies presented here, which span from broad-spectrum reviews to highly specific mechanistic investigations.

The path from preclinical discovery to clinical application, however, remains challenging. Key hurdles such as compound standardization, bioavailability, and the need for rigorous clinical validation persist, forming a well-documented translational gap [20]. The future of this field will undoubtedly rely on the seamless integration of advanced technologies to overcome these obstacles, including multi-omics approaches to comprehensively map bioactivity [21], artificial intelligence for accelerating target identification and deconvolution [22], and innovative drug delivery systems, such as nanotechnology, to enhance bioavailability and targeting [23,24]. Integrating these tools will be crucial to accelerate the translation of natural products into next-generation therapies.

This collection provides a valuable snapshot of a field in profound transformation, underscoring the immense potential held within nature’s pharmacopeia. To build on this momentum, we are delighted to announce that a second volume, “Natural Products for Interventions for Chronic Diseases: From Source to Therapy—2nd Edition”, is now open for submissions. We warmly invite the scientific community to continue this vital dialogue and contribute their latest findings, thereby collectively driving the discovery of next-generation therapies.

## Data Availability

No new data were created or analyzed in this study. Data sharing is not applicable to this article.
